# Inflammatory Cell Composition and Immune-Related microRNA Signature of Temporal Artery Biopsies From Patients With Giant Cell Arteritis

**DOI:** 10.3389/fimmu.2021.791099

**Published:** 2021-12-23

**Authors:** Luka Bolha, Alojzija Hočevar, Alen Suljič, Vesna Jurčić

**Affiliations:** ^1^ Institute of Pathology, Faculty of Medicine, University of Ljubljana, Ljubljana, Slovenia; ^2^ Department of Rheumatology, University Medical Centre Ljubljana, Ljubljana, Slovenia; ^3^ Faculty of Medicine, University of Ljubljana, Ljubljana, Slovenia; ^4^ Institute of Microbiology and Immunology, Faculty of Medicine, University of Ljubljana, Ljubljana, Slovenia

**Keywords:** giant cell arteritis, temporal artery biopsy, inflammatory infiltrate, inflammation, microRNA

## Abstract

**Objectives:**

The aim of this study was to quantitatively assess distinct immune cell subsets comprising inflammatory infiltrate in temporal artery biopsies (TABs) from patients with giant cell arteritis (GCA), and to link the obtained histopathological data with expression profiles of immune-related microRNAs (miRNAs).

**Methods:**

The study included 68 formalin-fixed, paraffin-embedded TABs from treatment-naïve patients, including 30 histologically positive GCA and 16 negative GCA TABs, and 22 control non-GCA TABs. Quantitative assessment of histological parameters was performed using histopathological and immunohistochemical techniques. miRNA expression analysis was performed by quantitative real-time PCR.

**Results:**

Intense transmural mononuclear inflammatory infiltrates in TAB-positive GCA arteries were predominantly composed of CD3^+^, CD4^+^ and CD8^+^ T lymphocytes, and CD68^+^ macrophages, accompanied by a strong nuclear overexpression of the nuclear factor of activated T cells, cytoplasmic 1 (NFATC) in the lymphocyte infiltrate fraction. Furthermore, TAB-positive GCA arteries were characterized by significant overexpression of nine pro-inflammatory miRNAs (miR-132-3p/-142-3p/-142-5p/-155-5p/-210-3p/-212-3p/-326/-342-5p/-511-5p) and a significant under-expression of six regulatory immune-related miRNAs (miR-30a-5p/-30b-5p/-30c-5p/-30d-5p/-30e-5p/-124-3p), whose expression levels significantly associated with most evaluated histopathological parameters. Notably, we revealed miR-132-3p/-142-3p/-142-5p/-155-5p/-212-3p/-511-5p as major promoters of arterial inflammation and miR-30a-5p/-30c-5p/-30d-5p as putative regulators of NFATC signaling in TAB-positive GCA arteries.

**Conclusion:**

Overall, we demonstrated that an altered arterial tissue-specific pro-inflammatory miRNA signature favors enhanced T cell-driven inflammation and macrophage activity in TAB-positive GCA arteries. Moreover, dysregulation of several immune-related miRNAs seems to contribute crucially to GCA pathogenesis, through impairing their regulatory activity towards T cell-mediated immune responses driven by the calcineurin (CaN)/NFAT signaling pathway, indicating their therapeutic, diagnostic and prognostic potential.

## Highlights

- NFATC is strongly overexpressed in GCA inflammatory infiltrates, predominantly comprising CD3^+^, CD4^+^ and CD68^+^ cells.
- Overexpressed miR-132-3p/-142-3p/-142-5p/-155-5p/-212-3p/-511-5p emerged as major promoters of T cell- and macrophage-driven inflammation in GCA lesions.
- Under-expression of miR-30a-5p/-30b-5p/-30c-5p/-30d-5p/-30e-5p/-124-3p may contribute to aberrant T cell functions driven by the CaN/NFAT signaling pathway.

## Introduction

Giant cell arteritis (GCA) is a systemic vasculitis affecting large- and medium-sized arteries, especially the extracranial branches of the carotid artery and the aorta (1). GCA is clinically characterized by ischemic symptoms and complications such as headache, jaw claudication, visual loss and ischemic stroke (2, 3) and systemic inflammation (3–5).

Histopathological changes in temporal artery biopsies (TABs) from GCA patients include a transmural, frequently granulomatous mononuclear inflammatory cell infiltrate, disruption of the internal elastic lamina and intimal thickening (2, 4). To date, there have been only limited studies focusing on a detailed characterization of the inflammatory infiltrate composition, which varied according to the presence of granulomas, B lymphocytes, CD8^+^ T lymphocytes and granulocytes (2, 3, 6–12). Moreover, there are no data on the temporal relationship between the composition and intensity of the TAB inflammatory infiltrate, duration of GCA and elevated levels of systemic inflammatory mediators and signs of inflammation in GCA patients, including erythrocyte sedimentation rate (ESR), C-reactive protein (CRP), acute phase serum amyloid A protein (A-SAA), peripheral blood thrombocytes, hemoglobin and fibrinogen (3, 5, 13).

The current concept of GCA pathogenesis suggests the initial activation of the *vasa vasorum* and resident vascular dendritic cells in the adventitia of the affected arteries, followed by infiltration, activation and differentiation of CD4^+^ T cells into IFN-γ-secreting Th1 and IL-17-secreting Th17 cells. Subsequently, monocytes are recruited to the arterial media, differentiating into macrophages and forming multinucleated giant cells (MGCs) (6, 8). In addition, IFN-γ induces the production of several chemokines and thus triggers the recruitment of CD8^+^ T cells (6, 9), whose implication and prognostic value in GCA have been determined previously, linking the intensity of the CD8^+^ T cell infiltrate in TABs with the severity of the disease (9). The response of CD8^+^ T, Th1 and Th17 cells to glucocorticoid therapy in GCA patients differs. Whereas glucocorticoids reduce the number of CD8^+^ T and Th17 cells in GCA lesions, Th1 cells persist in treated patients and are associated with chronically persistent vascular lesions (9, 12). In contrast to CD3^+^, CD4^+^ and CD8^+^ T cells and macrophages, the presence, intensity and significance of other inflammatory cells in TAB inflammatory infiltrate (e.g. CD20^+^ B cells and granulocytes) remains poorly investigated (4, 6, 8). However, two studies have linked the presence of B cell aggregates with the so-called artery tertiary lymphoid organs in the aorta (10) and TABs from GCA patients (11).

Several studies have suggested a prominent role of epigenetics in GCA pathogenesis (6, 14–16). DNA methylation analysis has revealed that most hypomethylated genes in GCA-affected TABs relate to aberrant T cell functions, promoted predominantly by the enhanced calcineurin (CaN)/nuclear factor of activated T cells (NFAT) signaling (16). Furthermore, dysregulation of microRNAs (miRNAs), a group of small non-coding RNAs involved in post-transcriptional regulation of gene expression, has been associated with inflammation and vascular remodeling in GCA (14, 15, 17). Our previous study on identifying miRNAs implicated in GCA pathogenesis (14) revealed several altered miRNAs, which have been previously associated with immune cell functions and pathways, including the miR-30 family, miR-124, the miR-132/212 cluster, miR-142, miR-155-5p, miR-210-3p, miR-326, miR-342-5p and miR-511-5p (18–30). Since there is currently no in-depth information available on dysregulated miRNAs that would relate to the distinct cellular composition of inflammatory infiltrates in TABs from GCA patients (2, 3), we performed a thorough quantitative histopathological evaluation of TABs from GCA and non-GCA patients, assessed GCA patients’ clinical data, and linked the obtained results with expression profiles of the selected aforementioned “immune-related” miRNAs. An important advantage of our study was the inclusion of TABs from treatment-naïve GCA patients, which enabled an unbiased insight into the pathogenesis of the disease.

## Materials and Methods

### Patients

The study included TABs from 46 clinically diagnosed treatment-naïve GCA patients, comprising 30 histologically positive and 16 negative TABs. The control non-GCA cohort included histologically negative TABs from 22 age-matched patients with a clinical suspicion of GCA, which was discarded after a complete patient work-up and follow-up. TABs were collected between September 2011 and December 2015, and GCA diagnosis established according to the American College of Rheumatology 1990 classification criteria (31). All patients were treatment-naïve prior to the TAB procedure. Notably, all enrolled patients had clinical suspicion of GCA and had undergone a TAB within 24 h from referral to the rheumatologist. Therapy commenced after performed TAB, when GCA diagnosis was proven. The study was performed in accordance with the Declaration of Helsinki and was approved by the National Medical Ethics Committee of the Republic of Slovenia [approval #65/01/17].

### Histopathology and Immunohistochemistry

For histopathological evaluation, hematoxylin and eosin staining (HE) of 4-µm thick sections of formalin-fixed, paraffin-embedded (FFPE) TABs was performed. All TABs were assessed histologically and demonstrated well preserved cellular detail. Approximately 10 HE sections of each TAB were examined. The following morphologically distinctive inflammatory cells were evaluated: MGCs, eosinophil and neutrophil granulocytes, and plasma cells. Lymphocyte subtypes, macrophages and cells expressing the NFAT, cytoplasmic 1 (NFATC) were identified immunohistochemically in a Ventana Benchmark automated slide stainer. Tissue sections were pre-treated with Cell Conditioning Solution 1 (CC1), and the following antibodies were used: CD3 (clone 2GV6, Ventana Medical Systems, Tucson, AZ, USA, ready to use antibody (RTU)); CD4 (clone SP35, Ventana Medical Systems, Tucson, AZ, USA, RTU); CD8 (clone SP57, Ventana Medical Systems, Tucson, AZ, USA, RTU); CD20 (clone L26, Dako, Denmark, dilution 1:4000); CD68 (clone PG-M1, Dako, Denmark, dilution 1:50) and NFATC (clone ab2796, Abcam, Cambridge, UK, dilution 1:50). Tissue sections were then treated with biotinylated secondary antibody, followed by incubation with peroxidase conjugated streptavidin (iVIEW™ DAB Detection Kit, Ventana Medical System, Tucson, AZ, USA). Visualization of the immunoreaction was achieved by using 3.3’-diaminobenzidine. Counterstaining was performed with hematoxylin. Immunostaining exhibited a brown nuclear reaction for NFATC and brown cytoplasmic reaction for other antibodies. Negative controls, omitting the primary antibodies, were included along with each run of samples.

Densities of CD3^+^, CD4^+^, CD8^+^, CD20^+^ and NFATC^+^ cells were assessed using an image analysis system (Cell and Tissue Analysis, Leica, Germany) and expressed as an average number of cells per mm^2^. CD68^+^ macrophages had indistinct borders and could not be counted in heavy infiltrates, so we adopted the following scoring system: 1 = fewer than 10 cells per mm^2^, 2 = 10–30 cells per mm^2^, 3 = heavy infiltrate involving < 50% arterial wall cross-sectional area, and 4 = heavy infiltrate involving > 50% arterial wall cross-sectional area. Due to the relatively small number of MGCs, eosinophil and neutrophil granulocytes, and plasma cells, we evaluated the maximal number of these cells per high power field (HPF; 400-fold magnification) using the following scoring system: 0 = no infiltrate, 1 = mild infiltrate (1–3 cells/HPF), 2 = moderate infiltrate (4–9 cells/HPF), and 3 = severe infiltrate (≥ 10 cells/HPF). In addition, we determined the CD4^+^:CD8^+^ T cell ratio and the percentage of CD20^+^ B cells from the total number of lymphocytes (T and B, meaning the sum of CD3^+^ and CD20^+^ cells, respectively). The NFATC^+^:CD4^+^ cell ratio was also determined from the obtained data.

### RNA Isolation

Total RNA was isolated from 10 10-µm thick sections of FFPE TAB samples with an AllPrep^®^ DNA/RNA FFPE Kit (80234, Qiagen, Germany), as described previously (14). Isolated RNA was stored at –80°C.

### Reverse Transcription and Quantitative Real-Time PCR

Reverse transcription was performed in 10 µl reaction volumes with the miRCURY LNA RT Kit (339340, Qiagen, Germany), as described previously (14). Quantitative real-time PCR (qPCR) analysis of miRNA expression was performed on a total of 46 TABs from treatment-naïve GCA patients and 22 TABs from non-GCA patient controls. qPCR was performed in 10 µl reaction mixtures on the Rotor-Gene Q real-time PCR cycler (Qiagen, Germany), with miRCURY SYBR Green PCR Kit (339347, Qiagen, Germany) and miRCURY LNA miRNA PCR Assays (339306; Qiagen, Germany), as previously described (14). Relative miRNA fold change was calculated using the 2^-ΔΔCt^ method (32). Fifteen candidate miRNAs were selected based on our previously performed miRNA expression profiling in TAB-positive GCA arteries (14) and literature mining focused on GCA-related immune responses, involving T and B cells, macrophages, inflammatory mediators and signaling pathways. Due to their prominent role in inflammation, according to the literature and relevant miRNA databases, we addressed the selected set of miRNAs as “immune-related” and/or “pro-inflammatory” throughout the manuscript. miRNA primer assays included in the analysis are listed in **Supplementary Table S1**.

### Computational Analysis

The miRDB database (33) and the STRING v11.0 online prediction tool (34) were used for identification of miRNA gene targets and to assess interactions between proteins of identified gene targets, respectively. Gene targets with miRDB target prediction scores 95–100 were included for analysis with the STRING online tool, where the highest confidence score (0.900) was used for all protein-protein interaction predictions.

### Statistical Analysis

Statistical analyses were performed with IBM SPSS Statistics 24.0 software (IBM Corporation, USA). To assess the normality of data distribution, the Q–Q plots, Kolmogorov-Smirnov and Shapiro-Wilk tests were used. Differences in histopathological, laboratory and clinical parameters, and relative miRNA expression levels between patient groups were statistically evaluated using the Mann-Whitney *U* test. Associations between patient characteristics and miRNA expression levels were evaluated with Spearman’s (*ρ*) correlation coefficients. A *p*-value of < 0.05 was considered statistically significant in all cases.

## Results

### Patients

Patient characteristics are presented in **Table 1**. Overall, there were no significant differences in patients’ gender, age at diagnosis or symptom duration time between TAB-positive and TAB-negative GCA and non-GCA patients (**Table 1**). There were also no significant differences in levels of evaluated clinical laboratory parameters, including ESR, CRP, platelet count, hemoglobin and fibrinogen, between TAB-positive and TAB-negative GCA patients (**Table 1**). A moderate significant correlation was found between levels of most laboratory parameters of GCA patients (**Supplementary Table S2**). Of evaluated clinical characteristics, we found significant differences only in the occurrence of jaw claudication between GCA patients with positive and negative TABs (**Table 1**).

**Table 1:** Patient characteristics.

Characteristic	GCA	GCA	Non-GCA	*p*-value^a^
	TAB-positive	TAB-negative	TAB-negative	
	(n = 30)	(n = 16)	(n = 22)	
Gender (male/female)	8/22	8/8	4/18	0.118
Age at diagnosis [years]	73 (55–89)	73 (57–92)	73 (58–87)	0.475
Symptom duration [days]^b^	30 (2–180)	21 (3–120)	21 (5–365)^c^	0.403
CD3^+^ T lymphocytes [cells/mm^2^]	226.7 (69.3–375)***	4.9 (1.3–10.3)***	1.3 (0–3.3)	<0.001
CD4^+^ T lymphocytes [cells/mm^2^]	159.3 (50.2–264.3)***	4.9 (1.7–11.5)***	1.6 (0–4)	<0.001
CD8^+^ T lymphocytes [cells/mm^2^]	96.5 (24.8–157.3)***	1.4 (0–3.3)**	0.1 (0–2)	<0.001
% CD8^+^ T lymphocytes^d^	35 (27.1–42.4)***	19.4 (0–30)	4.6 (0–38.5)	<0.001
CD4^+^:CD8^+^ T lymphocyte ratio	1.9 (1.4–2.7)***	3.3 (2.3–8.5)	5.7 (1.6–10)	<0.001
NFATC^+^ cells [cells/mm^2^]	176.6 (49.1–300.6)***	5.1 (1.2–14)***	0 (0–3)	<0.001
NFATC^+^:CD4^+^ cell ratio	1.1 (0.6–1.4)***	0.9 (0.6–1.6)***	0 (0–1)	0.062
CD20^+^ B lymphocytes [cells/mm^2^]	25.1 (3.5–167.5)***	0.8 (0–1.7)***	0 (0–0.3)	<0.001
% CD20^+^ B lymphocytes^e^	8.8 (3.1–29)***	9 (0–17.9)***	0 (0–7.6)	0.678
CD68^+^ macrophages [score]	3 (1–4)***	1 (1–1)**	0.5 (0–1)	<0.001
MGCs [score]	2.5 (0–4)***	0	0	<0.001
Eosinophil granulocytes [score]	2 (0–3)***	0	0	<0.001
ESR [mm/h]	84 (41–120)	78 (28–130)	NA	0.610
CRP [mg/ml]	71 (12–218)	50 (7–214)	NA	0.246
Platelets [10^9^/l]	364.5 (190–589)	321 (147–898)	NA	0.760
Hemoglobin [mg/ml]	122.5 (99–140)	119 (99–130)	NA	0.429
Fibrinogen [mg/ml]	7.9 (4.9–9.4)	8.4 (5.7–9.5)	NA	0.470
Constitutional symptoms; n (%)	23/30 (77)	11/16 (69)	NA	0.565
PMR; n (%)	6/30 (20)	3/16 (19)	NA	0.920
New headache; n (%)	26/30 (87)	11/16 (69)	NA	0.149
Jaw claudication; n (%)	17/30 (57)	0/16 (0)	NA	<0.001
Ischemic stroke; n (%)	0/30 (0)	0/16 (0)	NA	1.000
GCA relapse; n (%)	15/30 (50)	4/16 (25)	NA	0.116
Visual disturbances; n (%)	8/30 (27)	4/16 (25)	NA	0.903
Permanent visual loss; n (%)	2/30 (7)	0/16 (0)	NA	0.296

GCA, giant cell arteritis; TAB, temporal artery biopsy; ESR, erythrocyte sedimentation rate; CRP, C-reactive protein; MGC, multinucleated giant cell; PMR, polymyalgia rheumatica; NA, not applicable. Data are presented as median (range), unless otherwise specified. Data were evaluated using the Mann-Whitney U test. A p-value of < 0.05 was considered statistically significant. An asterisk indicates significance to the TAB-negative non-GCA group (**p < 0.01; ***p < 0.001).

a Statistical significance between GCA patient groups.

b Duration of symptoms prior GCA diagnosis.

c Duration of symptoms, which were discarded as GCA after a complete patient work-up and follow-up.

d Percentage of CD8^+^ T lymphocytes among all detected T lymphocytes constituting the inflammatory infiltrate.

e Percentage of CD20^+^ B lymphocytes among all lymphocytes constituting the inflammatory infiltrate.

### Histopathology

**Figure 1:**
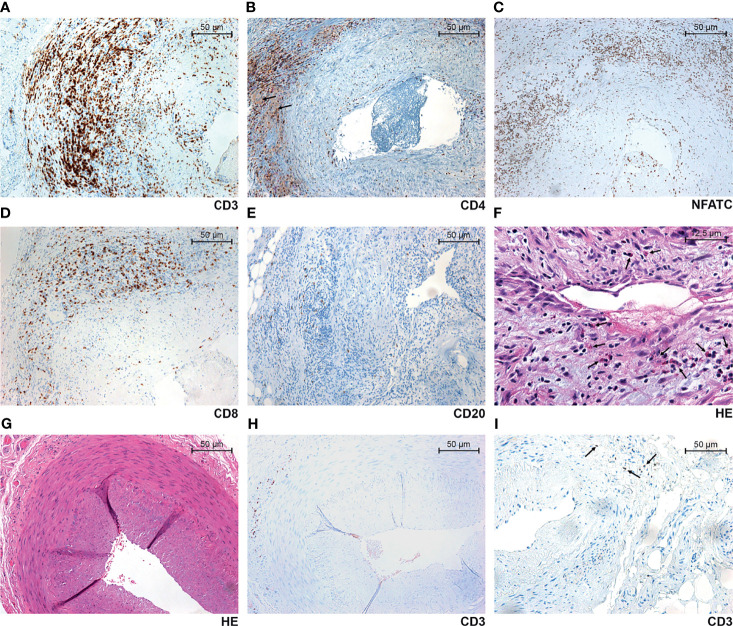
Selected representative images of TAB-positive GCA arteries **(A–F)**, TAB-negative GCA arteries **(G, H)** and non-GCA temporal arteries **(I)**. Prominent arterial wall infiltration with CD3^+^ **(A)**, CD4^+^ **(B)** and NFATC^+^ **(C)** cells. In addition to strong staining of lymphocytes, there was also a faint CD4 positivity of macrophages and multinucleated giant cells (arrows) **(B)**. In a majority of biopsies, a significant number of CD8^+^ lymphocytes **(D)**, relatively small number of CD20^+^ lymphocytes **(E)**, and a variable number of eosinophil granulocytes (arrows) **(F)** were found. Although not apparently present in hematoxylin and eosin (HE) stain **(G)**, some CD3^+^ lymphocytes could be found focally segmentally in the adventitia of TAB-negative GCA arteries **(H)**, and a very few in non-GCA temporal arteries of the control group (arrows) **(I)**.

All TAB-positive GCA arteries were characterized by a transmural mononuclear inflammatory cell infiltrate, which was most intense in the adventitia of 8/30 (27%) TABs and more evenly distributed in others. Overall, it was predominantly composed of lymphocytes and macrophages. MGCs were present in 26/30 (87%) TABs. Eosinophil and neutrophil granulocytes and plasma cells were present in 23/30 (77%), 19/30 (63%) and 21/30 (70%) TABs, respectively. One to three eosinophil granulocytes per HPF were focally found in four TABs, 4–9 in nine TABs and ≥ 10 in 10 TAB-positive GCA arteries (median value 20.5, range 10–34 in the latter group). In addition, 1–3 neutrophil granulocytes per HPF were present in 13 TABs and 4–9 in six TABs. No TAB-positive GCA arteries were characterized by ≥ 10 neutrophil granulocytes per HPF. Intimal hyperplasia and disruption of the internal elastic lamina were present in all TAB-positive GCA arteries. Immunohistochemically, there was a predominance of CD3^+^ and CD4^+^ T lymphocytes and CD68^+^ macrophages in the TAB-positive GCA group. In addition, NFATC was highly expressed in lymphocytes and the number of NFATC^+^ cells was slightly higher compared to CD4^+^ T lymphocytes. No transmural mononuclear inflammatory cell infiltrates were present in TAB-negative GCA and non-GCA arteries, with no MGCs or eosinophil and neutrophil granulocytes. Although small numbers of lymphocytes and macrophages were detected in these two groups using immunohistochemistry, they were apparently absent in HE-stained slides. The median percentage of CD8^+^ T cells among all detected T lymphocytes constituting the inflammatory infiltrate was 35% in TAB-positive GCA arteries, 19.4% in TAB-negative GCA arteries and 4.6% in TAB-negative non-GCA arteries (**Table 1**), and was significantly higher in the TAB-positive GCA group, compared to the TAB-negative groups (*p* < 0.001). The percentage of CD20^+^ B cells comprising the lymphocyte infiltrate fraction was significantly higher in TABs from GCA patients (median 8.8–9.0%), compared to the controls (median 0.0%) (*p* < 0.001), although with no significant differences between GCA patient groups (*p* = 0.678) (**Table 1**). All assessed histopathological parameters in TABs from GCA and non-GCA patients are presented in **Table 1** and selected representative images of immune cell subsets comprising the inflammatory infiltrate in TAB-positive GCA arteries in **Figure 1**.

### Interrelation Between Histopathological and Laboratory Parameters in GCA Patients

Spearman’s *ρ* correlation analysis revealed a strong significant correlation between most evaluated histopathological parameters (**Table 2**). The strongest significant positive correlation emerged between the numbers of infiltrated CD3^+^, CD4^+^, CD8^+^ and NFATC^+^ cells (all *ρ* > 0.940; *p* < 0.001), indicating a strong interrelationship among these immune cells in GCA lesions. A strong significant positive correlation was also determined between the numbers of CD3^+^, CD4^+^ and CD20^+^ cells, and between scores of MGCs and CD68^+^ cells (all *ρ* > 0.902; *p* < 0.001) (**Table 2**). Notably, the CD4^+^:CD8^+^ T lymphocyte ratio showed a negative correlation with all evaluated histopathological parameters, and had the strongest significant negative correlation with the number of CD8^+^ T cells and scores of CD68^+^ cells (both *ρ* = –0.812; *p* < 0.001) (**Table 2**).

**Table 2:** Spearman’s correlation matrix of associations between histopathological parameters in TABs from GCA patients.

	CD3^+^	CD4^+^	CD8^+^	CD4^+^:CD8^+^	NFATC^+^	NFATC^+^:CD4^+^	CD20^+^	CD68^+^	MGCs	Eosinophils
	[cells/mm^2^]	[cells/mm^2^]	[cells/mm^2^]	ratio	[cells/mm^2^]	ratio	[cells/mm^2^]	[score]	[score]	[score]
CD3^+^ [cells/mm^2^]	1									
CD4^+^ [cells/mm^2^]	0.988***	1								
CD8^+^ [cells/mm^2^]	0.971***	0.954***	1							
CD4^+^:CD8^+^ ratio	–0.717***	–0.660***	–0.812***	1						
NFATC^+^ [cells/mm^2^]	0.959***	0.964***	0.947***	–0.694***	1					
NFATC^+^:CD4^+^ ratio	0.292*	0.273	0.332*	–0.241	0.477**	1				
CD20^+^ [cells/mm^2^]	0.907***	0.902***	0.897***	–0.698***	0.882***	0.329*	1			
CD68^+^ [score]	0.846***	0.835***	0.884***	–0.812***	0.852***	0.365*	0.790***	1		
MGCs [score]	0.846***	0.830***	0.865***	–0.781***	0.861***	0.435**	0.785***	0.923***	1	
Eosinophils [score]	0.739***	0.724***	0.777***	–0.724***	0.743***	0.364*	0.619***	0.786***	0.881***	1

MGC, multinucleated giant cell. Spearman’s correlation coefficients (ρ) between evaluated histopathological parameters in TABs from 46 GCA patients, including 30 TAB-positive and 16 TAB-negative GCA patients. A p-value of < 0.05 was considered statistically significant (*p < 0.05; **p < 0.01; ***p < 0.001).

In addition, scores of MGCs and eosinophil granulocytes significantly associated with CRP levels, with *ρ* = 0.313 (*p* = 0.041) and *ρ* = 0.344 (*p* = 0.024), respectively (**Table 3**). Overall, there were no other significant associations between the evaluated histopathological and laboratory parameters of GCA patients and the symptom duration of GCA (**Table 3**).

**Table 3:** Spearman’s correlation matrix of associations between symptom duration, histopathological and laboratory parameters of GCA patients.

Histopathological parameter	GCA symptom duration	ESR	CRP	Platelets	Hemoglobin	Fibrinogen
	[days]	[mm/h]	[mg/ml]	[10^9^/l]	[mg/ml]	[mg/ml]
CD3^+^ [cells/mm^2^]	0.144	–0.050	0.195	0.048	0.164	–0.011
CD4^+^ [cells/mm^2^]	0.186	–0.036	0.204	0.044	0.176	–0.007
CD8^+^ [cells/mm^2^]	0.068	0.040	0.239	0.113	0.088	0.130
CD4^+^:CD8^+^ ratio	–0.046	0.076	–0.263	–0.003	0.025	–0.132
NFATC^+^ [cells/mm^2^]	0.161	0.027	0.213	0.085	0.184	0.041
NFATC^+^:CD4^+^ ratio	–0.079	0.190	0.209	0.188	0.106	0.280
CD20^+^ [cells/mm^2^]	0.100	–0.143	0.084	0.032	0.191	0.078
CD68^+^ [score]	0.091	0.024	0.248	0.128	0.038	0.220
MGCs [score]	0.074	0.062	0.313*	0.134	0.090	0.113
Eosinophils [score]	–0.110	0.108	0.344*	0.130	0.177	0.091

ESR, erythrocyte sedimentation rate; CRP, C-reactive protein; MGC, multinucleated giant cell. Spearman’s correlation coefficients (ρ) between evaluated parameters of 46 GCA patients, including 30 TAB-positive and 16 TAB-negative GCA patients. A p-value of < 0.05 was considered statistically significant (*p < 0.05) (marked in bold).

### Pro-Inflammatory miRNAs Are Overexpressed in Inflamed GCA Temporal Arteries

**Figure 2:**
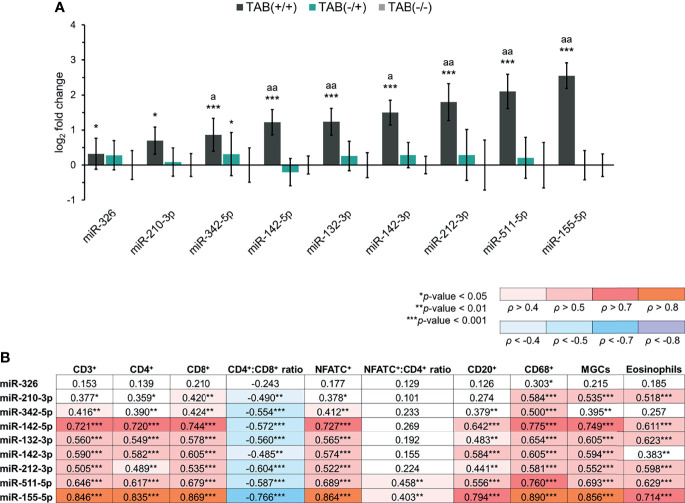
Expression of pro-inflammatory miRNAs in TABs from GCA and non-GCA patients. **(A)** Expression of pro-inflammatory miRNAs in TAB-positive GCA arteries [TAB(+/+), n = 30], TAB-negative GCA arteries [TAB(-/+), n = 16] and non-GCA temporal arteries [TAB(-/-), n = 22]. The bars represent the means of log_2_ fold change values ± S.D. Data were evaluated using the Mann-Whitney *U* test. An asterisk indicates significance to the TAB(-/-) group (*p < 0.05; ***p < 0.001) and the letter a the significance between the TAB(+/+) and TAB(-/+) groups (^a^ *p* < 0.01; ^aa^ *p* < 0.001). **(B)** Association (Spearman’s *ρ* correlation coefficients) between miRNA expression levels and evaluated histopathological parameters in TABs from GCA patients (n = 46). A *p*-value of < 0.05 was considered statistically significant in all cases.

We determined a significant 1.3- to 5.9-fold overexpression of the nine selected “pro-inflammatory” miRNAs (miR-132-3p/-142-3p/-142-5p/-155-5p/-210-3p/-212-3p/-326/-342-5p/-511-5p) in TAB-positive GCA arteries (all *p* ≤ 0.043), compared to non-GCA controls (**Figure 2A**). Expression levels of miR-132-3p/-142-3p/-142-5p/-155-5p/-212-3p/-342-5p/-511-5p were also significantly higher in TAB-positive GCA arteries, compared to TAB-negative GCA arteries (all *p* ≤ 0.006) (**Figure 2A**). Of the nine miRNAs, miR-342-5p was the only miRNA significantly overexpressed in the TAB-negative GCA group (*p* = 0.04), compared to the controls. Expression profiles of other pro-inflammatory miRNAs did not differ between TAB-negative arteries from GCA and non-GCA patients (**Figure 2A**).

### Pro-Inflammatory miRNA Signature Associates With Arterial Wall Immunopathology

When assessing the interrelation between pro-inflammatory miRNA expression levels and quantitatively assessed histopathological parameters in TABs from GCA patients, we found a significant positive correlation between the number of infiltrated CD3^+^, CD4^+^, CD8^+^, NFATC^+^ and CD20^+^ immune cells and expression levels of miR-142-5p/-132-3p/-142-3p/-212-3p/-511-5p/-155-5p (all *ρ* > 0.440; *p* < 0.01) (**Figure 2B**). Moreover, these six miRNAs, together with miR-210-3p/-342-5p, also significantly positively correlated with scores of CD68^+^ macrophages, MGCs and eosinophil granulocytes (all *ρ* > 0.380; *p* < 0.01). Notably, the CD4^+^:CD8^+^ T lymphocyte ratio showed a negative correlation with expression levels of all assessed pro-inflammatory miRNAs and miR-155-5p showed the strongest negative correlation (*ρ* = –0.766; *p* < 0.001) (**Figure 2B**). The NFATC^+^:CD4^+^ cell ratio significantly positively correlated only with expression levels of miR-511-5p/-155-5p (**Figure 2B**). Overall, expression of miR-155-5p and miR-142-5p showed the strongest Spearman’s *ρ* correlation with 9/10 and 7/10 evaluated histopathological parameters, respectively (**Figure 2B**).

Of the nine pro-inflammatory miRNAs, only miR-326 significantly positively associated with platelet levels in GCA patients. There were no other significant associations between miRNA expression levels, evaluated laboratory parameters and symptom duration of GCA (**Supplementary Table S3**).

### Altered Expression of the miR-30 Family and miR-124 in Relation to the CaN/NFAT Signaling Pathway and TAB Histopathological Features in GCA

**Figure 3:**
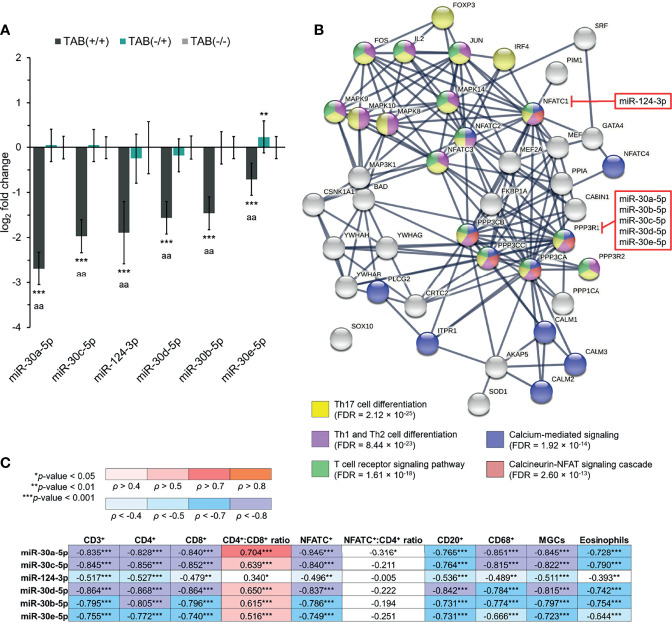
The miR-30 family and miR-124 in relation to the CaN/NFAT signaling pathway in GCA. **(A)** Expression of miR-30 family members and miR-124-3p in TAB(+/+) [n = 30], TAB(-/+) [n = 16] and TAB(-/-) [n = 22] groups. The bars represent the means of log_2_ fold change values ± S.D. Data were evaluated using the Mann-Whitney *U* test. An asterisk indicates significance to the TAB(-/-) group (**p < 0.01; ***p < 0.001) and the letter a the significance between the TAB(+/+) and TAB(-/+) groups (^aa^ *p* < 0.001). **(B)** STRING association network and Gene Ontology biological process annotation of top predicted functional partners of the CaN (PPP3CA, PPP3CB, PPP3CC, PPP3R1, PPP3R2) and NFAT (NFATC1, NFATC2, NFATC3, NFATC4) family members, involved in the CaN/NFAT signaling pathway. Individual proteins and protein–protein interactions with the highest confidence score (0.900) are presented. miRNAs predicted to target the *PPP3R1* and *NFATC1* genes are indicated in red squares. **(C)** Association (Spearman’s *ρ* correlation coefficients) between miRNA expression levels and evaluated histopathological parameters in TABs from GCA patients (n = 46). A *p*-value of < 0.05 was considered statistically significant in all cases. FDR, false discovery rate.

Our previous study on miRNA expression profiling in GCA arterial lesions (14) revealed several altered miRNAs that could interrelate with the induction of the CaN/NFAT signaling pathway, which plays a central role in T cell-mediated immune responses (35, 36) and is also involved in GCA pathogenesis (16). Specifically, we focused on the miR-30 family and miR-124, both previously implicated in CaN/NFAT signaling (28, 30). In the present study, we found a significant 1.6- to 6.4-fold under-expression of miR-30a-5p/-30b-5p/-30c-5p/-30d-5p/-30e-5p and miR-124-3p (all *p* < 0.001) in TAB-positive GCA arteries, compared to non-GCA controls (**Figure 3A**). Similarly, all miRNAs showed a significant under-expression in TAB-positive GCA arteries (all *p* < 0.001), compared to TAB-negative GCA arteries. As determined, miR-30e-5p was the only significantly differentially expressed miRNA in TAB-negative GCA arteries, compared to the non-GCA controls, and was overexpressed 1.2-fold (*p* = 0.004) (**Figure 3A**).

By utilizing the miRDB database, we showed that miR-30a-5p/-30b-5p/-30c-5p/-30d-5p/-30e-5p and miR-124-3p are predicted to target the *PPP3R1* and *NFATC1* genes, respectively (**Figure 3B**). Both PPP3R1 and NFATC1 are among key components of the CaN/NFAT signaling pathway (35, 36). Accordingly, STRING association network and Gene Ontology biological process annotation revealed that PPP3R1 and NFATC1, together with their top predicted functional partners, strongly associate with Th17, Th1 and Th2 cell differentiation, T cell receptor signaling pathway, calcium-mediated signaling and the CaN/NFAT signaling cascade (**Figure 3B**).

Notably, the expression of miR-30a-5p/-30b-5p/-30c-5p/-30d-5p/-30e-5p and miR-124-3p negatively associated with 9/10 evaluated histopathological parameters in TABs from GCA patients, excluding the CD4^+^:CD8^+^ T lymphocyte ratio, which showed a significant positive association with all assessed miRNAs (**Figure 3C**). As determined, expression levels of miR-30a-5p/-30c-5p/-30d-5p showed the strongest negative correlation with 7/10 evaluated histopathological parameters (all *ρ* < –0.760; *p* < 0.001), including the number of infiltrated CD3^+^, CD4^+^, CD8^+^, NFATC^+^, CD20^+^ and CD68^+^ immune cell subsets and MGCs, and also the strongest positive correlation with the CD4^+^:CD8^+^ T lymphocyte ratio (all *ρ* > 0.630; *p* < 0.001) (**Figure 3C**). Compared to the miR-30 family, miR-124-3p expression levels showed weaker Spearman’s *ρ* correlation with all the evaluated histopathological parameters (**Figure 3C**). Overall, there were no significant associations among miR-30 family and miR-124 expression profiles, symptom duration of GCA and evaluated laboratory parameters of GCA patients (**Supplementary Table S3**).

### Differences in Histopathological Parameters and miRNA Expression Levels Between GCA Patients Suffering Headache and Jaw Claudication

To assess clinical significance of TAB immunopathology and miRNA alterations in GCA patients, we compared the evaluated histopathological features and miRNA expression levels between GCA patients experiencing constitutional symptoms, patients with polymyalgia rheumatica (PMR) and patients suffering headache, jaw claudication, GCA relapse and visual disturbances. As determined, patients with headache had a significantly higher number of CD8^+^ T lymphocytes present in their TAB inflammatory infiltrate and an increased expression of miR-142 (both miR-142-3p/-5p) (**Table 4**). Notably, inflammatory infiltrate in TABs from GCA patients suffering jaw claudication was characterized by a significantly higher number of CD3^+^, CD4^+^, CD8^+^, NFATC^+^, CD20^+^ and CD68^+^ inflammatory cells, accompanied by a significantly lower expression of miR-30a-5p/-30b-5p/-30c-5p/-30d-5p/-30e-5p/-124-3p and a significantly higher expression of miR-142-5p/-155-5p/-212-3p, compared to patients without jaw claudication (**Table 4**). There were no significant differences in histopathological parameters and miRNA expression between GCA patients experiencing constitutional symptoms, PMR, GCA relapse and visual disturbances (**Table 4**).

**Table 4:** Histopathological parameters and miRNA expression levels in TABs from GCA patients with different clinical characteristics.

	Constitutional symptoms		PMR		Headache		Jaw claudication		GCA relapse		Visual disturbances	
	Yes	No	Yes	No	Yes	No	Yes	No	Yes	No	Yes	No
	(n = 34)	(n = 12)	(n = 9)	(n = 37)	(n = 37)	(n = 9)	(n = 17)	(n = 29)	(n = 19)	(n = 27)	(n = 12)	(n = 34)
CD3^+^ [cells/mm^2^]	120.5 (1.3–375)	197.9 (3–329.1)	116 (1.3–325)	139.7 (1.3–375)	182.5 (1.3–375)	10 (1.3–218.3)	235 (69.3–375)**	10 (1.3–340)	125 (1.3–312.7)	139.7 (1.3–375)	161.7 (3.4–321)	132.4 (1.3–375)
CD4^+^ [cells/mm^2^]	89.3 (1.7–264.3)	138.8 (3.8–230.9)	86.1 (1.7–264.3)	103.6 (1.7–260.2)	127.3 (1.7–264.3)	11 (1.7–157.2)	161.4 (50.2–264.3)**	11 (1.7–230.9)	92.5 (1.7–243.2)	119 (1.7–264.3)	121.2 (3.4–230.9)	98.1 (1.7–264.3)
CD8^+^ [cells/mm^2^]	45.1 (0–148.8)	81.5 (0–157.3)	42 (0.2–126)	66 (0–157.3)	71 (0–157.3)*	3.3 (0–81.5)	90.3 (24.8–157.3)**	2.5 (0–148)	45.2 (0–148)	55 (0–157.3)	58.1 (0–117.8)	50.1 (0–157.3)
CD4^+^:CD8^+^ ratio	2.1 (1.5–8.5)	2 (1.4–5.9)	2.1 (1.5–8.5)	2 (1.4–4.6)	2 (1.4–5.9)	2.2 (1.6–8.5)	2 (1.4–2.7)	2.2 (1.4–8.5)	1.9 (1.4–4.6)	2.2 (1.4–8.5)	2.2 (1.7–5.9)	2 (1.4–8.5)
NFATC^+^ [cells/mm^2^]	83 (1.2–300.6)	146.8 (3.5–288.9)	79 (1.7–227.9)	121.3 (1.2–300.6)	140.6 (1.2–300.6)	8.3 (1.7–180.9)	152.9 (49.1–300.6)**	8.3 (1.2–262.7)	87 (1.2–296.3)	140.6 (1.7–300.6)	100.2 (2–296.3)	108.4 (1.2–300.6)
NFATC^+^:CD4^+^ ratio	1 (0.6–1.6)	1.2 (0.7–1.6)	1 (0.7–1.4)	1.1 (0.6–1.6)	1 (0.6–1.6)	1 (0.6–1.4)	1 (0.6–1.4)	1 (0.6–1.6)	1.2 (0.6–1.5)	1 (0.6–1.6)	0.9 (0.6–1.4)	1.1 (0.6–1.6)
CD20^+^ [cells/mm^2^]	10.8 (0–167.5)	15.7 (0.3–60.2)	13 (0.3–51)	11 (0–167.5)	13 (0–167.5)	1.7 (0–47)	18 (3.5–167.5)*	1.3 (0–71.3)	18 (0–69)	10 (0–167.5)	14.5 (0.3–71.3)	12 (0–167.5)
CD68^+^ [score]	2 (1–4)	2.5 (1–4)	2 (1–3)	2 (1–4)	2 (1–4)	1 (1–4)	2 (1–4)*	1 (1–4)	2 (1–4)	2 (1–4)	2 (1–4)	2 (1–4)
MGCs [score]	1 (0–4)	2.5 (0–4)	1 (0–3)	1 (0–4)	2 (0–4)	0 (0–4)	2 (0–4)	0 (0–4)	1 (0–4)	1 (0–4)	1.5 (0–4)	1 (0–4)
Eosinophils [score]	0.5 (0–3)	1 (0–3)	0 (0–3)	1 (0–3)	1 (0–3)	0 (0–3)	2 (0–3)	0 (0–3)	1 (0–3)	0 (0–3)	0.5 (0–3)	0.5 (0–3)
miR-30a-5p	–2 (–3.9 to 0.6)	–1.9 (–4.2 to 0.7)	–2.4 (–3.3 to 0.1)	–2 (–4.2 to 0.7)	–2.1 (–4.2 to 0.7)	–0.5 (–3.3 to 0.2)	–2.5 (–3.6 to –0.3)**	–0.6 (–4.2 to 0.7)	–2.3 (–4.2 to 0.3)	–2 (–3.9 to 0.7)	–2 (–3.6 to 0.7)	–2.1 (–4.2 to 0.6)
miR-30b-5p	–1.2 (–2.8 to 0.4)	–0.7 (–2.4 to 0.3)	–1 (–2.1 to 0)	–1.1 (–2.8 to 0.4)	–1.3 (–2.8 to 0.4)	–0.4 (–1.4 to 0.2)	–1.4 (–2.8 to 0)**	–0.3 (–2.4 to 0.4)	–1.3 (–2.8 to 0.3)	–1 (–2.4 to 0.4)	–1.3 (–2.4 to 0.1)	–1 (–2.8 to 0.4)
miR-30c-5p	–1.7 (–2.7 to 0.5)	–1.9 (–2.9 to 0.4)	–1.8 (–2.4 to –0.1)	–1.8 (–2.9 to 0.5)	–1.8 (–2.9 to 0.5)	–0.6 (–2 to 0.3)	–2.2 (–2.7 to –0.2)**	–0.3 (–2.9 to 0.5)	–1.8 (–2.6 to 0.4)	–1.7 (–2.9 to 0.5)	–1.7 (–2.9 to 0.4)	–1.8 (–2.7 to 0.5)
miR-30d-5p	–1.2 (–2.5 to 0.4)	–1.4 (–2 to 0.3)	–1.2 (–2.4 to –0.2)	–1.4 (–2.5 to 0.4)	–1.4 (–2.5 to 0.4)	–0.6 (–1.8 to –0.1)	–1.6 (–2.5 to –0.3)*	–0.6 (–2 to 0.4)	–1.4 (–2.5 to 0)	–1.1 (–2.4 to 0.4)	–1.5 (–2.1 to 0.3)	–1.1 (–2.5 to 0.4)
miR-30e-5p	–0.5 (–1.4 to 0.7)	–0.6 (–1 to 0.4)	–0.4 (–1 to 0.4)	–0.6 (–1.4 to 0.7)	–0.7 (–1.4 to 0.7)	0 (–0.8 to 0.4)	–0.7 (–1.4 to 0.2)**	0 (–1.3 to 0.7)	–0.6 (–1.4 to 0.4)	–0.4 (–1.1 to 0.7)	–0.7 (–1.1 to 0.4)	–0.4 (–1.4 to 0.7)
miR-124-3p	–1.9 (–3.8 to 1)	–1.3 (–2.9 to 0.4)	–1.8 (–2.9 to 0.5)	–1.7 (–3.8 to 1)	–1.8 (–3.8 to 1)	–0.8 (–2.1 to 1)	–2 (–3.8 to 0.1)*	–1 (–2.9 to 1)	–1.7 (–2.7 to 0.7)	–1.8 (–3.8 to 1)	–2 (–2.9 to 0.5)	–1.6 (–3.8 to 1)
miR-132-3p	1 (–0.3 to 2.1)	0.8 (–0.6 to 2)	0.6 (–0.2 to 1.6)	1.1 (–0.6 to 2)	1.1 (–0.6 to 2.1)	0.7 (0.3–1.8)	1.2 (0–2.1)	0.7 (–0.6 to 2.1)	1.1 (–0.3 to 2.1)	0.7 (–0.6 to 2.1)	0.9 (–0.6 to 2.1)	1 (–0.3 to 2.1)
miR-142-3p	1.4 (–1.7 to 2.7)	1.3 (–3.8 to 2)	1.8 (–0.4 to 2.4)	1.3 (–3.8 to 2.7)	1.5 (–3.8 to 2.7)*	0.3 (–0.4 to 1.8)	1.5 (0.1–2.7)	1.3 (–3.8 to 2.1)	1.3 (–0.4 to 2.4)	1.5 (–3.8 to 2.7)	1.5 (–3.8 to 2.4)	1.4 (–1.7 to 2.7)
miR-142-5p	0.9 (–1.6 to 2.2)	1.2 (–3.9 to 1.9)	1.1 (–1.2 to 1.9)	0.9 (–3.9 to 2.2)	1.1 (–3.9 to 2.2)*	–0.3 (–1.2 to 1.8)	1.3 (–0.3 to 2.2)*	0.8 (–3.9 to 1.9)	0.8 (–0.6 to 2.1)	1 (–3.9 to 2.2)	1 (–3.9 to 2.1)	0.9 (–1.6 to 2.2)
miR-155-5p	1.8 (–0.3 to 3.9)	1.9 (–1 to 3.6)	1.8 (–1 to 3.1)	2 (–0.8 to 3.9)	2.2 (–1 to 3.9)	0.5 (0–3.1)	2.6 (0.9–3.7)*	0.6 (–1 to 3.9)	2.3 (–0.4 to 3.6)	1.8 (–1 to 3.9)	1.7 (–1 to 3.4)	2 (–0.4 to 3.9)
miR-210-3p	0.4 (–1.2 to 3.6)	0.6 (–1.7 to 2.7)	–0.1 (–1.7 to 2)	0.6 (–1.2 to 3.6)	0.6 (–1.7 to 3.6)	0.3 (–1 to 2.3)	0.6 (–1.7 to 2.6)	0.4 (–1.1 to 3.6)	0.3 (–1 to 2.7)	0.6 (–1.7 to 3.6)	0.6 (–0.8 to 2.6)	0.4 (–1.7 to 3.6)
miR-212-3p	1.6 (–0.7 to 3)	1.2 (–0.1 to 3.3)	1.1 (0–1.8)	1.4 (–0.7 to 3.3)	1.5 (–0.7 to 3.3)	1 (0–2.8)	1.8 (0.4–3)*	1.1 (–0.7 to 3.3)	1.8 (–0.6 to 3.3)	1.1 (–0.7 to 3)	1.2 (0–2.8)	1.5 (–0.7 to 3.3)
miR-326	0.3 (–0.5 to 2.2)	0.2 (–1.2 to 0.9)	0.2 (–0.5 to 0.8)	0.3 (–1.2 to 2.2)	0.3 (–1.2 to 2.2)	0.1 (–0.5 to 0.5)	0.3 (–0.5 to 1.1)	0.3 (–1.2 to 2.2)	0.2 (–0.5 to 1.1)	0.3 (–1.2 to 2.2)	0.2 (–1.2 to 0.7)	0.3 (–0.5 to 2.2)
miR-342-5p	0.7 (–0.7 to 2.2)	0.3 (–0.3 to 2)	0.5 (–0.2 to 1.4)	0.7 (–0.7 to 2.2)	0.7 (–0.3 to 2.2)	0.3 (–0.7 to 1.1)	0.9 (0–2)	0.3 (–0.7 to 2.2)	0.5 (–0.7 to 2)	0.6 (–0.3 to 2.2)	0.2 (–0.2 to 2)	0.7 (–0.7 to 2.2)
miR-511-5p	1.2 (–1.4 to 4.4)	1.5 (–1.7 to 3.6)	1 (0.2–3)	1.4 (–1.7 to 4.4)	1.4 (–1.7 to 4.4)	0.9 (–0.6 to 2.4)	1.7 (–0.3 to 4.4)	0.9 (–1.7 to 4.4)	1.4 (–0.6 to 4.4)	1.2 (–1.7 to 4.4)	1.2 (–1.7 to 4.4)	1.3 (–0.6 to 4.4)

GCA, giant cell arteritis; MGC, multinucleated giant cell; PMR, polymyalgia rheumatica. Data are presented as median (range). Log_2_ fold change values are presented for each miRNA. Data were evaluated using the Mann-Whitney U test. Analysis was performed on a total of 46 TABs from GCA patients, including 30 TAB-positive and 16 TAB-negative GCA patients. A p-value of < 0.05 was considered statistically significant. An asterisk indicates significance between GCA patient groups (*p < 0.05; **p < 0.01) (marked in bold).

## Discussion

This study links the composition of inflammatory cell infiltrate with immune-related miRNA signature in affected temporal arteries from GCA patients, and emphasizes the significance of miRNA dysregulation in impaired regulation of arterial inflammation in GCA, particularly in relation to NFATC expression and promotion of T cell functions through the CaN/NFAT signaling pathway. To the best of our knowledge, we performed the first quantitative histopathological and immunohistochemical analysis of inflammatory infiltrate in inflamed GCA, non-inflamed GCA and non-GCA TABs, in which distinct immune cell subsets were related to altered arterial miRNA profiles. By including TABs from treatment-naïve patients, we aimed to get an unbiased insight into the arterial wall inflammatory histological features and miRNA profiles underlying GCA-related inflammation, without treatment-induced immunosuppression associated with GCA remission (6, 37).

GCA is primarily considered a T cell-mediated disease (4, 6, 8, 9, 38), and the affected TAB-positive GCA arteries are usually histologically characterized by a transmural granulomatous inflammatory infiltrate with T lymphocytes, macrophages and MGCs, disruption of the internal elastic lamina and intimal thickening (2, 3). Our quantitative histological assessment corresponded to these classical histological GCA features in the TAB-positive GCA group, revealing abundant numbers of CD3^+^, CD4^+^, CD8^+^, NFATC^+^ and CD68^+^ cells, and a variable number of CD20^+^ B lymphocytes, MGCs and eosinophil granulocytes, compared to TAB-negative GCA arteries, where small numbers of CD3^+^, CD4^+^ and CD68^+^ cells were focally segmentally detectable by immunohistochemistry only. Notably, elevated numbers of CD20^+^ B cells suggested the presence of the so-called artery tertiary lymphoid organs, lymphoid aggregates with well-defined compartmentalization of B cells, T cells and follicular dendritic cells, which form at sites of chronic inflammation in peripheral non-lymphoid organs (10, 11, 39), present mostly in the media of GCA-affected TABs (11, 39). We found similar structures in only two TAB-positive GCA arteries, with a high percentage of CD20^+^ B cells (18% and 29%), which coincided with a longer symptom duration prior to biopsy in these patients (75 and 120 days, respectively). Eosinophil and neutrophil granulocytes were a common finding, present in 23/30 (77%) and 19/30 (63%) of TAB-positive GCA arteries, respectively. Contrary to the low number of neutrophil granulocytes, a significant number of eosinophil granulocytes was focally present in 10/30 (33%) of TABs, mostly in those with prominent granulomatous inflammation, thus resembling the composition of Th2 cell-induced granulomas. Eosinophil granulocytes, together with MGCs, were also significantly associated with CRP levels in our GCA cohort. Other studies, dealing with the composition of inflammatory infiltrate in GCA, commonly included patients already on glucocorticoid treatment, which is generally known to diminish eosinophil granulocytes. In these studies, eosinophils in TABs were described as scant (2, 40) or rarely present (2, 7), except in initial biopsies (40). Overall, the role of eosinophil granulocytes in GCA pathogenesis has not been well-established. In inflammation, these cells can cause severe tissue damage by releasing inflammatory mediators, they can exacerbate T cell responses and are attracted to the site of inflammation by different cytokines and chemokines produced by other inflammatory cells and/or endothelial cells (41).

The percentage of CD8^+^ T cells among all detected T lymphocytes constituting the inflammatory infiltrate in our TAB-positive GCA group was 35%, which fitted within the 12–46% CD8^+^ T cell range in GCA-affected TABs reported previously (42). Nonetheless, relatively high numbers of infiltrated CD8^+^ T cells in TAB-positive GCA arteries probably resulted in a significantly lower tissue CD4^+^:CD8^+^ T lymphocyte ratio (1.9), compared to TAB-negative GCA (3.3) and non-GCA temporal arteries (5.7), and indicated a more severe disease course in TAB-positive GCA patients (6, 9). Notably, numbers of CD8^+^ T lymphocytes were significantly higher in TAB inflammatory infiltrates of patients suffering headache and jaw claudication from our GCA patient cohort (**Table 4**).

It is generally recognized that miRNAs play a key role in maintaining immune homeostasis and normal immune function, whereas miRNA dysregulation may lead to the breakage of immune tolerance and the development of autoimmune diseases (43, 44). Several studies have shed light on the significance of miRNA dysregulation in GCA pathogenesis, revealing their notable role in regulating the vascular smooth muscle cell phenotype that underlies arterial remodeling and intimal hyperplasia, and their interaction with gene pathways involved in regulation of the immune system, the ubiquitin-proteasome system and the RNA-induced silencing complex (14, 15). The data obtained from our previous work on identifying miRNAs involved in GCA pathogenesis (14) served as a basis for the selection of candidate immune-related miRNAs, whose expression profiles were thoroughly assessed and related to evaluated histopathological parameters in TABs from GCA and non-GCA patients in the present study. We included the miR-30 family, miR-124, the miR-132/212 cluster, miR-142, miR-155-5p, miR-210-3p, miR-326, miR-342-5p and miR-511-5p, which have been previously associated with T cell immune responses, macrophage activity and pro-inflammatory cytokine signaling (18–30).

We showed that the nine selected pro-inflammatory miRNAs (miR-132-3p/-142-3p/-142-5p/-155-5p/-210-3p/-212-3p/-326/-342-5p/-511-5p) were significantly overexpressed only in TAB-positive GCA arteries, characterized by an intense mixed inflammatory infiltrate. Significant positive association between their expression levels and evaluated histopathological parameters suggested that induction of these miRNAs relates to a distinct cellular composition of GCA inflammatory infiltrates within arterial walls, predominantly T lymphocytes and macrophages. Since the composition of inflammatory infiltrates may significantly differ between GCA patients and lead to different clinical outcomes (2, 3), immune-related miRNAs could be used as markers to discriminate these patients.

Notably, association analysis indicated that miR-142-5p and miR-155-5p, together with miR-132-3p/-142-3p/-212-3p/-511-5p, are major promoters of T cell-driven inflammation and macrophage activity in TAB-positive GCA arteries (**Figure 2**). Our indications were supported by the fact that miR-155 overexpression in activated T cells promotes autoimmune inflammation through enhancing T cell activation and proliferation, production and release of pro-inflammatory cytokines, polarization of Th cells into IFN-γ-secreting Th1 and IL-17-secreting Th17 phenotypes, and induction of macrophage inflammatory responses (20, 21, 45), mechanisms that are related to GCA pathogenesis (4, 6, 8). Furthermore, upregulation of miR-142-5p and miR-551 enhances macrophage polarization into the M2 phenotype (26, 46), which is also significantly increased in inflamed GCA arteries and involved in tissue repair and remodeling (47). Since miR-155-5p and miR-142-5p, both target the suppressor of cytokine signaling 1 (SOCS1) gene (45, 46), these two miRNAs may promote vascular inflammation and remodeling in GCA lesions through affecting the SOCS1 signaling. However, further studies are needed to confirm such implication of these two miRNAs in GCA pathogenesis. It has been determined that induction of the miR-132/212 cluster (miR-132 and miR-212) positively associates with enhanced differentiation and activation of Th17 cells, and elevated IL-17 production (18). Similarly, upregulation of miR-142-3p in activated CD3^+^ T lymphocytes promoted IL-1β-mediated signaling (48), which is under the influence of the IL-6–IL-17 cytokine cluster crucially involved in Th17 differentiation in GCA arterial lesions (4). Taken together, overexpression of the miR-132/212 cluster and miR-142-3p in TAB-positive GCA arteries, may relate to the pathogenic pathways mediated by the Th17 T cell lineage. Nonetheless, aforementioned interrelation needs to be confirmed in GCA by further in-depth studies.

The strong interrelationship between evaluated immune cell subsets, primarily between infiltrated CD3^+^, CD4^+^, CD8^+^ and NFATC^+^ cells, implied that T cell-mediated immune responses in TAB-positive arteries from our GCA patient cohort were driven by the dysregulated CaN/NFAT signaling pathway. In response to inflammatory stimuli, the CaN/NFAT signaling cascade initiates by the influx of extracellular calcium, which binds to calmodulin and thus activates the CaN-mediated dephosphorylation of NFAT, resulting in its translocation into the nucleus, where NFAT promotes expression of genes related to T cell development, activation and differentiation (16, 35, 36). Due to its prominent role in regulating T cell functions, dysregulation of the calcium-mediated CaN/NFAT signaling pathway frequently associates with the development of autoimmune diseases (35), including GCA (16).

Our analysis revealed that strong nuclear overexpression of NFATC, detected in immune cells in TAB-positive GCA arteries, probably resulted from the upstream induction of CaN, mediated through impaired regulatory activity of the miR-30 family towards the CaN regulatory subunit PPP3R1. A strong significant negative association between elevated numbers of CD3^+^, CD4^+^, CD8^+^ and NFATC^+^ cells, and significant under-expression of miR-30 family members, predominantly miR-30a-5p/-30c-5p/-30d-5p, in TAB-positive GCA arteries supported this hypothesis (**Figure 3**). Since miRNAs from the miR-30 family target critical components of the calcium/CaN signaling pathway in podocytes, including *TRPC6*, *PPP3CA*, *PPP3CB*, *PPP3R1* and *NFATC3* (28), dysregulated miR-30 expression might be essential for aberrant T cell-mediated immune responses in GCA arterial lesions. Furthermore, our results suggest that deregulated induction of NFATC [alias NFATC1 (36)] in immune cell infiltrate within TAB-positive GCA arteries might be directly mediated by under-expressed miR-124, whose inhibitory function towards NFATC has been determined previously (30). To confirm this hypothesis, however, additional functional studies are needed. In addition, the significant negative association between scores of CD68^+^ macrophages and MGCs, and altered miRNA expression levels in TAB-positive GCA arteries (**Figure 3**), corresponded to the impaired inhibitory activity of the miR-30 family and miR-124 towards pro-inflammatory macrophage functions (49, 50). Overall, the pro-inflammatory microenvironment in vessel walls of GCA-affected temporal arteries seems to be strongly influenced by epigenetic promotion of T cell functions through the CaN/NFAT signaling pathway, mediated by DNA methylation (16) and alterations in miRNA expression, both evidently contributing to GCA pathogenesis.

Despite including TABs from treatment-naïve GCA patients, the limited number of included patients represents the main limitation of our current single-center study. Moreover, there is a need for further detailed phenotypic characterization and quantitative assessment of immune cell subpopulations comprising inflammatory infiltrate in GCA arterial lesions and linking them with their specific miRNA fingerprints. Such miRNA-based molecular characterization of specific arterial histopathological features might discriminate patients in terms of their heterogeneous cellular inflammatory infiltrate composition and aid in anticipating GCA clinical outcome. Furthermore, additional information on dysregulated miRNAs and their target gene networks in peripheral blood mononuclear cells and granulocytes, and their interrelation with tissue and circulatory inflammatory mediators, would provide valuable new insights into the complexity of GCA pathogenesis.

In summary, our study provides novel information on miRNA involvement in the vascular immunopathology of GCA. We showed that altered arterial tissue-specific immune-related miRNA profiles associate with enhanced T cell-driven inflammation and macrophage activity in TAB-positive GCA arteries, and probably promote T cell-mediated immune responses through dysregulation of the CaN/NFAT signaling pathway. Our results indicate a potential role of dysregulated CaN/NFAT signaling and aberrant T cell functions in GCA-related ischemic events (i.e. headache and jaw claudication), where an altered expression of miR-30a-5p/-30b-5p/-30c-5p/-30d-5p/-30e-5p/-124-3p/-142-3p/-142-5p/-155-5p/-212-3p might also play a role. Nevertheless, further studies are needed to identify these miRNAs as potential novel therapeutic targets, and to prove their diagnostic and prognostic potential in GCA, including their utilization on biological fluids such as blood serum and plasma.

## Data Availability Statement

All data relevant to the study are included in the article or uploaded as **Supplementary Material**.

## Ethics Statement

The studies involving human participants were reviewed and approved by The National Medical Ethics Committee of the Republic of Slovenia [approval #65/01/17]. Written informed consent for participation was not required for this study in accordance with the national legislation and the institutional requirements.

## Author Contributions

LB: qPCR analysis, data analysis, data interpretation, manuscript drafting and writing. AH: provision of clinical data and a specialist advice. AS: morphometric analysis of TABs, data analysis, manuscript writing. VJ: case selection of patients and TABs, histopathological assessment of TABs, data interpretation, manuscript drafting and writing. All authors read, participated in improving and approved the final version of the manuscript.

## Funding

This work was supported by the Slovenian Research Agency [research core funding No. P3-0054].

## Conflict of Interest

The authors declare that the research was conducted in the absence of any commercial or financial relationships that could be construed as a potential conflict of interest.

## Publisher’s Note

All claims expressed in this article are solely those of the authors and do not necessarily represent those of their affiliated organizations, or those of the publisher, the editors and the reviewers. Any product that may be evaluated in this article, or claim that may be made by its manufacturer, is not guaranteed or endorsed by the publisher.
