# Supplementary material for: Inflammatory Cell Composition and Immune-Related microRNA Signature of Temporal Artery Biopsies From Patients With Giant Cell Arteritis
Source: Front Immunol. 2021 Dec 23;12:791099. doi: 10.3389/fimmu.2021.791099 (PMC8733475; doi:10.3389/fimmu.2021.791099)
Supplement: Supplementary file 1 [file DataSheet_1.docx]

***Supplementary Material***

**SUPPLEMENTARY TABLES**

**Supplementary Table S1.** miRNA primer assays.

| miRNA | Catalogue number^a^ |
| --- | --- |
| miR-30a-5p | YP00205695 |
| miR-30b-5p | YP00204765 |
| miR-30c-5p | YP00204783 |
| miR-30d-5p | YP00206047 |
| miR-30e-5p | YP00204714 |
| miR-124-3p | YP00206026 |
| miR-132-3p | YP00206035 |
| miR-142-3p | YP00204291 |
| miR-142-5p | YP00204722 |
| miR-155-5p | YP00204308 |
| miR-210-3p | YP00204333 |
| miR-212-3p | YP00204170 |
| miR-326 | YP00204512 |
| miR-342-5p | YP00204516 |
| miR-511-5p | YP00204046 |
| miR-27a-5p^b^ | YP00203901 |
| miR-362-3p^b^ | YP00204394 |
| miR-500a-5p^b^ | YP00203904 |
| UniSp6^c^ | YP00203954 |

*^a^miRCURY LNA miRNA PCR Assay catalogue number (product number 339306, Qiagen, Germany).*

*^b^miRNA primer assay used as an endogenous control for data normalization.*

*^c^miRNA primer assay used as an interplate calibrator.*

**Supplementary Table S2.** Association between evaluated clinical laboratory parameters of GCA patients.

|  | ESR | CRP | Platelets | Hemoglobin | Fibrinogen |
| --- | --- | --- | --- | --- | --- |
|  | [mm/h] | [mg/ml] | [10^9^/l] | [mg/ml] | [mg/ml] |
| ESR [mm/h] | 1 |  |  |  |  |
| CRP [mg/ml] | 0.615*** | 1 |  |  |  |
| Platelets [10^9^/l] | 0.661*** | 0.505** | 1 |  |  |
| Hemoglobin [mg/ml] | –0.501** | –0.247 | –0.431** | 1 |  |
| Fibrinogen [mg/ml] | 0.499** | 0.399* | 0.383 | –0.301 | 1 |

*ESR, erythrocyte sedimentation rate; CRP, C-reactive protein. Spearman’s correlation coefficients (ρ) between evaluated clinical laboratory parameters of 46 GCA patients, including 30 TAB-positive and 16 TAB-negative GCA patients. A p-value of < 0.05 was considered statistically significant (*p < 0.05; **p < 0.01; ***p < 0.001).*

**Supplementary Table S3.** Association between miRNA expression levels and evaluated laboratory parameters of GCA patients.

|  | GCA symptom duration | ESR | CRP | Platelets | Hemoglobin | Fibrinogen |
| --- | --- | --- | --- | --- | --- | --- |
| miRNA | [days] | [mm/h] | [mg/ml] | [10^9^/l] | [mg/ml] | [mg/ml] |
| miR-30a-5p | –0.064 | 0.045 | –0.172 | 0.025 | –0.109 | 0.002 |
| miR-30b-5p | –0.008 | 0.039 | –0.205 | 0.001 | –0.113 | 0.040 |
| miR-30c-5p | –0.024 | 0.052 | –0.157 | 0.011 | –0.171 | 0.027 |
| miR-30d-5p | –0.060 | 0.163 | –0.040 | 0.146 | –0.240 | 0.039 |
| miR-30e-5p | –0.039 | 0.063 | –0.074 | 0.095 | –0.213 | 0.209 |
| miR-124-3p | 0.117 | 0.200 | 0.022 | 0.176 | –0.157 | 0.369 |
| miR-132-3p | 0.240 | 0.097 | 0.234 | 0.226 | –0.015 | 0.130 |
| miR-142-3p | 0.129 | –0.131 | 0.054 | –0.097 | 0.103 | 0.129 |
| miR-142-5p | 0.114 | –0.023 | 0.208 | 0.036 | 0.162 | 0.220 |
| miR-155-5p | 0.160 | 0.002 | 0.242 | 0.066 | 0.121 | 0.088 |
| miR-210-3p | 0.005 | 0.051 | 0.227 | 0.073 | –0.033 | 0.373 |
| miR-212-3p | 0.179 | –0.031 | 0.219 | 0.046 | –0.032 | 0.087 |
| miR-326 | 0.033 | 0.244 | 0.186 | 0.400** | –0.276 | 0.262 |
| miR-342-5p | 0.204 | –0.171 | 0.175 | 0.128 | –0.042 | –0.041 |
| miR-511-5p | –0.041 | 0.138 | 0.123 | 0.246 | –0.140 | 0.044 |

*ESR, erythrocyte sedimentation rate; CRP, C-reactive protein. Spearman’s correlation coefficients (ρ) between evaluated parameters of 46 GCA patients, including 30 TAB-positive and 16 TAB-negative GCA patients. A p-value of < 0.05 was considered statistically significant (**p < 0.01).*
